# Lifetime Management of Aortic Stenosis: Evolving Strategies and Personalized Decision-Making

**DOI:** 10.3390/jcm15062269

**Published:** 2026-03-17

**Authors:** Christina M. Mansour, Long-Co L. Nguyen, Fabio Sagebin, Antonio H. Frangieh

**Affiliations:** 1 Mary & Steve Wen Cardiovascular Division, University of California Irvine, Irvine, CA 92697, USA; 2 Division of Cardiac Surgery, University of California Irvine, Irvine, CA 92697, USA

**Keywords:** TAVR, SAVR, valve durability, lifetime management, index procedure

## Abstract

**Background**: The landscape surrounding aortic stenosis continues to evolve as transcatheter aortic valve replacement (TAVR) is increasingly performed in younger and lower-risk patients who are likely to outlive their index prosthesis. With the rapid evolution in management of aortic stenosis, treatment has shifted from a single-procedure paradigm toward a lifetime strategy that anticipates future reinterventions. Therefore, having a foundational understanding and a thoughtful strategy when selecting the index procedure is paramount. **Objectives**: The objectives of this review article are to review contemporary evidence and provide a structured framework for lifetime management of severe AS. We focus on optimizing index valve selection and planning durable, safe pathways for subsequent reinterventions. **Conclusions**: Lifetime management of aortic stenosis requires a forward-looking, patient-centered Heart Team approach that extends beyond immediate procedural success. Strategic selection of the index valve intervention is crucial, and this is guided by anatomy, life expectancy, comorbidities, patient preference, and future reintervention feasibility. Together, these factors are essential to optimize long-term outcomes. As treatment paradigms continue to evolve, anticipatory planning and advanced simulation technologies will play an increasingly central role in delivering durable personalized care for patients with severe AS.

## 1. Introduction

Aortic stenosis (AS) is the most prevalent valvular heart disease in developed countries, and the incidence increases exponentially with age. Though several etiologies, including congenital abnormalities and rheumatic heart disease, lead to AS, age-related degeneration is the most common. In the United States alone, calcific aortic valve disease affects an estimated 2.7 million adults ≥75 years, with ~700,000 meeting criteria for severe AS. In Europe and the U.S., the prevalence of severe AS among those ≥75 years is ~3.4% [1]. Once symptoms develop, prognosis without intervention is poor. Contemporary guidelines agree that valve replacement guided by Heart Team-based shared decision-making is the only therapy that improves survival [2,3].

Over the past decade, rapid advances in TAVR have broadened treatment eligibility from inoperable and high-risk patients to selected low-risk cohorts. The initial studies of TAVR were conducted in inoperable and high-risk patients, which limited detection of late valve degeneration as many died before valve dysfunction occurred. The latest evidence shows promising durability of TAVR valves beyond 5 years and freedom from structural valve deterioration at 10 years of duration [4,5,6]. Additionally, although SAVR is recommended by ACC/AHA guidelines for patients with severe AS aged < 65 years, the analysis of the Vizient Clinical Data Base demonstrates a 2.7-fold increase in TAVR utilization in patients aged < 65 years during the study period of 2015 to 2021, reaching nearly equal volumes to SAVR by 2021 (47.5% TAVR vs. 52.5% SAVR; *p* = NS) [2,7]. Concurrently, Transcatheter Valve Therapy (TVT) Registry data show that the mean age of low-risk TAVR recipients fell below 75 years in 2020 and is likely to decline further, as current AHA/ACC guidelines recommend shared decision-making between SAVR and TAVR for patients 65 to 80 years old [2,8].

These findings and trends have shifted practice from a “single procedure” mindset to a lifetime management framework. Rather than asking only *whether* and *when* to intervene, clinicians must plan *how* today’s choice shapes tomorrow’s options for their patients. Contemporary guidelines anchor choice of intervention in a Heart Team process that integrates patient characteristics (age, estimated life expectancy, quality of life, values, ability to tolerate anticoagulation, and concomitant conditions), access and valve anatomy (including transfemoral [TF] feasibility and calcification patterns), procedural risk, and repeat procedure options [2,3]. In our review we discuss current outstanding issues on lifetime management of severe aortic stenosis and go into the key considerations in optimizing planning the index aortic valve intervention as well as subsequent reinterventions.

To address these issues, we performed a comprehensive review of contemporary guidelines, randomized trials across surgical risk populations, large registries from the United States and Europe, and key meta-analyses. Our synthesis is guided by a multidisciplinary, patient-centered Heart Team approach, including our institutional experience. This review emphasizes lifetime management with a focus on contemporary data and modern transcatheter technologies. We propose a patient-centered framework for the management of severe aortic stenosis that emphasizes strategic planning of both initial and subsequent valve interventions.

## 2. The Role of Surgical Intervention as a Foundation of Lifetime Aortic Stenosis Management

**Figure 1:**
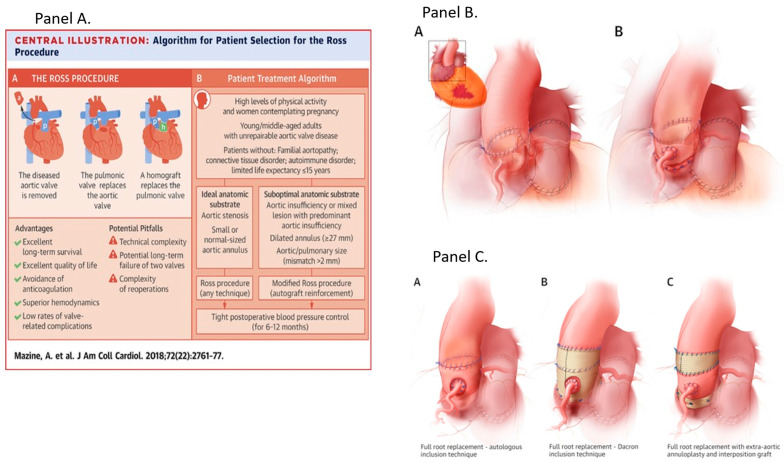
The Ross Procedure: Panel (**A**) details a summary of patient selection and key surgical approaches for the Ross operation. Ideal candidates are younger, active patients with favorable aortic anatomy. The pulmonary autograft in the aortic position can be performed with two different techniques. A: The Ross procedure process is shown along with the strengths and limitations. B: Indications, patient selection, and contraindications for the Ross procedure. Panel (**B**) illustrates the subcoronary technique. A: Subcoronary technique. B: Full root replacement technique. Panel (**C**) illustrates full root replacement techniques along with modern reinforcement strategies designed to reduce late autograft dilation. A: Autologous reinforcement technique; B: Dacron reinforcement technique; C: Extra-aortic annuloplasty with interposition graft. Adapted with permission from Ref. [13] under license from Elsevier and Copyright Clearance Center in 2026.

**Figure 2:**
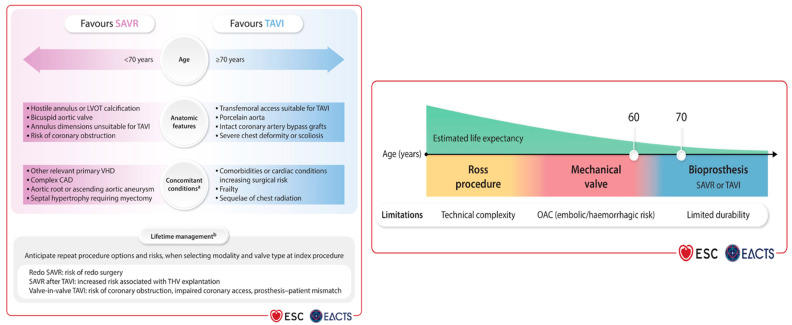
The 2025 ESC/EACTS Valvular Heart Disease Guidelines section on management of aortic stenosis. The 2025 ESC/EACTS developed updated valvular guidelines that detail key factors influencing the initial choice between SAVR, TAVI, mechanical valves, bioprostheses, and the Ross procedure. Decision-making incorporates age, anatomy, comorbidities, technical feasibility, and lifetime valve planning. Adopted from 2025 ESC/EACTs Valvular Heart Disease. (^a^ The original figure states that relative contraindications to TAVR are LV thrombus and infective endocarditis. ^b^ The original figure indicates that lifetime management for aortic stenosis is especially important for patients in whom anticipated life expectancy is thought to exceed the durability of the valve.) Ref. [3] with permission under license from Oxford University Press License in 2026.

Currently, there is no ideal aortic valve substitute. The Ross procedure, which involves replacing the aortic valve with the patient’s own pulmonary valve and placing a pulmonary valve homograft in the pulmonary position, is the only operation that has shown restoration of life expectancy to age-matched general population in young patients with AS (Figure 1). Some studies have shown a survival rate of >80% up to 20 years after surgery [9]. However, both the pulmonary homograft and the neo-aortic valve are at risk of degeneration over time, necessitating the potential need for reintervention on two valves. The reported cumulative risk of aortic and/or pulmonary reintervention after the Ross procedure is 1.2% per year, which lies between that of biological and mechanical AVR [10]. The Ross procedure is a Class IIb indication for young adults requiring aortic valve surgery despite demonstrating improved long-term survival compared to both mechanical and bioprosthetic AVR and higher freedom from valve-related complications compared with bioprosthetic AVR [2,10]. Of note, advantages may be limited by the technical expertise of the operator as most available data are based on single-center or single-surgeon experience. This complex procedure should be offered selectively to younger patients with appropriate anatomy and tissue characteristics, especially when they are not a candidate for anticoagulation, and be performed only at comprehensive valve centers by experienced surgeons. The balance between life expectancy and prosthetic valve durability varies continuously across the age range, with more durable valves preferred for patients with a longer life expectancy (Figure 2). In patients < 50 years of age, a mechanical valve is suggested as the prosthesis of choice (Class 2a) given their exceptional durability, with a reintervention rate of approximately 0.5% per patient per year [2,11]. In general, mechanical AVR is also preferred in patients 50 to 70 years old, with low bleeding risk due to its superior durability, albeit at the cost of lifelong anticoagulation [12]. On the other hand, bioprosthetic valves have become the dominant choice across most age groups, driven by continual improvements in design and leaflet technology. They offer a compelling alternative, particularly in older patients or when anticoagulation is contraindicated or undesirable, despite their greater susceptibility to structural degeneration over time. Mechanical and bioprosthetic valves are recommended with an equal level of evidence in patients aged 50 to 65 years (Class 2a); however most patients undergoing valve intervention in Europe and North America receive a bioprosthetic valve either by SAVR or TAVR [2,3].

Current ACC/AHA and ESC guidelines (Figure 2) recommend TAVR as the preferred treatment option for older patients with a life expectancy >1 year or those considered at high surgical risk (Society of Thoracic Surgeons Predicted Risk of Mortality or EuroSCORE II > 8%). However, guidelines are open-ended between SAVR and TAVR for patients older than 65 years.

Over the past decade, with the wide-spread use of TAVR, improved diagnostic techniques, and growing indications for intervention, there has been a substantial increase in the number of patients undergoing AVR, including younger patients who are most likely to outlive their prosthetic valve. In the US population, median survival in patients 65–69, 70–79, and ≥80 years of age undergoing isolated AVR is 13, 9, and 6 years, respectively. For AVR plus coronary artery bypass grafting (CABG) procedures, median survival is lower at 10, 8, and 6 years, respectively. Only 5% of isolated AVR patients had a high STS perioperative risk of mortality ≥ 10%; among this cohort, median survival was 2.5–2.7 years [14]. Assuming bioprosthetic valve durability of 12 to 15 years for patients with longer life expectancy, at least one additional aortic valve procedure is expected.

## 3. Index Intervention: Key Considerations for Choosing TAVR vs. SAVR

**Figure 3:**
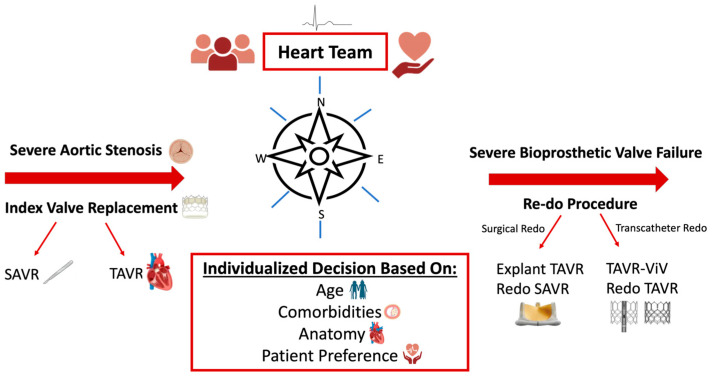
Lifetime Management Framework for Initial and Re-do Aortic Valve Interventions in Severe Aortic Stenosis. Deliberate planning of the index procedure is fundamental to lifetime management in severe aortic stenosis. This figure illustrates a lifetime management approach to severe aortic stenosis: emphasizing importance of index procedure, integrating Heart Team assessment, key anatomic and clinical variables, and ultimately patient preference. Initial SAVR or TAVR informs re-intervention strategies such as TAVR ViV, Redo TAVR, redo SAVR, or TAVR explant. Original figure created by the authors of this paper.

From a lifetime-management perspective, selecting the initial intervention is critical as it must provide the greatest long-term durability but also permit pathways for second and potentially third reinterventions (Figure 3). Accordingly, we will review the key anatomical, clinical, and procedural considerations that must be weighed in patients whose life expectancy is likely to exceed the anticipated durability of the implanted prosthesis. Importantly, no single strategy is appropriate for every patient; rather, the optimal approach must be individualized. In addition, center expertise and operator experience significantly influence outcomes and should be incorporated into decision-making, alongside a thorough discussion of patient values and preferences, to ensure alignment with long-term goals of care.

### 3.1. Durability

Randomized control trials have shown showed non-inferiority of TAVR compared with SAVR in patients of all risk profiles, with comparable longer-term outcomes demonstrated during follow-up periods up to 10 years. Five-year results from PARTNER 3 and Evolut Low Risk show broadly comparable clinical outcomes between TAVR and SAVR in low-risk patients [4,15]. And more recently, longer-term follow-up from the PARTNER 3 trial data shows that SAVR and TAVR continue to provide similar clinical outcomes in low-risk patients with symptomatic severe aortic stenosis at 7 years [5]. Additionally, data from the NOTION trial demonstrated that risk of major clinical outcomes was not different 10 years after treatment. Furthermore, the risk of severe bioprosthesis structural valve deterioration (SVD) was lower after TAVR compared with SAVR [6]. Although mid-to long-term durability data for contemporary TAVR prostheses are encouraging, it is important to recognize that these data are largely derived from older, higher risk populations with limited life expectancy and lower cumulative lifetime hemodynamic stress. Extrapolation of these findings to younger, more active patients remains uncertain. Younger patients may face a substantially longer period of exposure to structural valve deterioration, repeat interventions, and coronary access challenges, underscoring the need for cautious interpretation of current durability data and for a lifetime management approach rather than a single-procedure-focused strategy.

### 3.2. Coronary Obstruction and Coronary Artery Access

**Figure 4:**
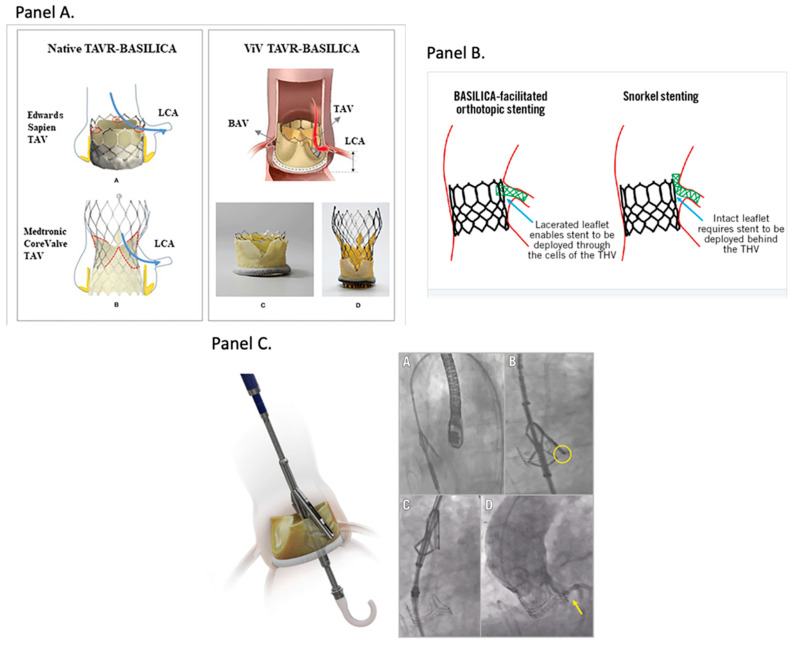
Alternative Approaches When Aortic Valve Explant is Not an Option. In patients with high-risk anatomy and prohibitive surgical risk, adjunctive strategies are the next best step. These include techniques such as BASILICA leaflet laceration, Pi-Cardia leaflet modification, or chimney stenting. Panel (**A**) shows a schematic illustration of BASILICA performed in native aortic stenosis where intentional leaflet laceration prior to transcatheter valve deployment prevents sinus sequestration and preserves coronary flow. Additionally, in degenerated bioprosthetic valves, leaflet laceration facilitates safe valve-in-valve TAVR by preventing displacement of the leaflet against the sinotubular junction and coronary ostia, reducing the risk of coronary obstruction. Panel (**B**) shows successful leaflet laceration that allows for coronary stents to be deployed orthotopically through the transcatheter heart valve frame (THV), thus maintaining native coronary access. In the absence of leaflet modification, coronary protection may require stent deployment behind the THV frame, resulting in snorkel configuration. Panel (**C**) shows a fluoroscopic demonstration of leaflet laceration and preserved coronary flow. A and B show positioning arm and splitting element, respectively. In B, the Pi-Cardia ShortCut device shown in the yellow circle is splitting the bioprosthetic leaflet facing the main coronary artery. In C, the splitting is occurring. In D, the yellow arrow is showing good coronary flow post-split and TAVR impanation indicating patency of the left main coronary artery. These alternative approaches are crucial to allow for a possibility for a redo valve procedure. All images are adapted from the respective journals with permission obtained. Of note, the panels are adapted under license obtained from the respective journals and reproduced from open-access source under Frontiers Refs. [27,28,29,30,31] in 2026. (Panel (**A**): A&B [31]; C&D [29,30]; Panel (**B**) [28]; Panel (**C**) [27]).

Coronary artery obstruction is a rare (incidence of <1%) but devastating complication with a 50% mortality rate at 30 days [16]. In native-valve TAVR, low coronary heights, shallow SOV, low STJ height, elongated leaflets, bulky calcified nodules, and small projected distances between a virtual valve implant and the coronary ostium, SOV, and STJ increase the risk of coronary obstruction [17]. Pragmatic cut-offs widely used in practice include coronary height < 10–12 mm, SOV diameter < 30 mm, a virtual THV-to-coronary (VTC) distance < 4 mm, valve-specific residual sinus diameter < 5 mm, cusp height < coronary height, and leaflet calcium volume < 600 mm^3^ [18,19]. Adequate dimensions and clearances support a TAVR-first approach, with deliberate commissural alignment, if possible, and implant depth to safeguard future coronary access [20]. In redo settings, particularly TAVR-in-TAVR, patients with effaced sinuses or small STJ height and diameter have a high risk of coronary obstruction, especially if a high frame valve is used. A very small valve-to-aorta (VTA) clearance at the STJ (<2 mm) may signal risk for sinus sequestration and jeopardized re-access [21,22]. In the case of Evolut-in-Evolut, VTA distance is additionally measured if the leaflets extend above the STJ, and VTA of <2 mm is similarly considered high-risk for sinus sequestration [23]. Low heights, small sinuses, or critical clearances generally favor SAVR first if surgical risk is acceptable. In patients with long life expectancy, aortic root replacement can be considered to facilitate later TAVR-in-SAVR. However, if risk of aortic root surgery is not acceptable, mechanical AVR can be considered in patients with anatomies not compatible with TAVR-in-SAVR [24]. In patients with high-risk anatomy and prohibitive surgical risk, mitigating maneuvers such as BASILICA leaflet laceration, Pi-Cardia leaflet modification, and chimney stenting can be considered in the context of index or redo-procedures (Figure 4) [25,26,27].

### 3.3. Aortic Annulus Size and Patient Prosthesis Mismatch

The presence of a small aortic annulus (SAA) is a frequent finding that poses a considerable challenge in the management of patients with severe aortic stenosis. The prevalence of patients with a SAA varies from 17% to 44%, and predominately comprises older women. While there is no consensus definition of what qualifies as a small aortic annulus, many propose an annular diameter of <21–23 mm [32]. Patients with an SAA undergoing aortic valve replacement are at increased risk of patient–prosthesis mismatch (PPM), where the prosthetic valve is functioning normally but provides an effective orifice area (EOA) that is disproportionately small for the patient’s body size [33]. PPM occurs at an indexed EOA (EOAi) of less than 0.85 cm^2^/m^2^, and those patients with an EOAi less than 0.65 cm^2^/m^2^ are classified as severe. PPM is associated with high residual gradients, structural valve deterioration, higher mortality risk, and less left ventricular mass regression after AVR [34,35]. Annulus enlargement is recommended during the index SAVR to prevent patient–prosthesis mismatch following the index procedure. Additionally, patients with a small prosthesis are at higher risk for sinus sequestration, coronary obstruction, and mortality with future valve reinterventions [36]. Data from observational studies and sub-studies from randomized trials suggest the rate of severe PPM is higher in SAVR than for TAVR in patients with SAA [37,38]. The difference is largely attributed to the ability to oversize a transcatheter heart valve (THV) relative to the patient’s annulus size given its lack of a sewing ring and ability for supra-annular leaflet positioning. However, patients with SAA receive TAVR still up to a 20% rate of severe PPM [39]. Furthermore, oversizing THVs in patients with SAA who also have heavy asymmetric annular and LVOT calcifications can increase the risk of annular rupture and coronary obstruction [40].

Balloon-expandable valve (BE THV) data from the PARTNER trials show no adverse effect of small annular size or female sex on hard clinical outcomes or mid-term valve durability. Substudies demonstrate that patients with small annuli have comparable survival, stroke risk, hemodynamics, and structural valve deterioration compared with broader TAVR cohorts. Collectively, these analyses support consistent BEV performance across these higher-risk subgroups [41,42]. Compared to BE THV, self-expanding valves (SE THV) tend to have larger EOA, lower post-procedural mean gradients, and lower PPM rates [43]. In the Small Annuli Randomized to Evolut or SAPIEN Trial (SMART), which evaluated patients with an annulus area < 430 mm^2^, the 1-year incidence of moderate or severe PPM was 11.2% with SE THV versus 35.3% with BE THV (*p* < 0.001). Although overall clinical composite outcomes were comparable, self-expanding valves demonstrated superior hemodynamic performance [44]. Longer-term follow-up will be needed to determine whether these hemodynamic advantages ultimately translate into meaningful differences in clinical outcomes.

Although TAVR generally demonstrates lower rates of severe PPM compared with SAVR, some studies suggest that young age may increase the likelihood of severe PPM even after TAVR [45,46]. Younger patients tend to have higher cardiac output requirements and larger metabolic demands, which can render a given effective orifice area insufficient despite technically successful TAVR. Surgical strategies proposed to mitigate PPM in young patients with SAA include stentless valve implantation (offers larger effective orifice areas by eliminating a rigid stent frame), sutureless valve implantation (permits larger internal diameters), aortic root or annular enlargement procedures, and complete aortic root replacement [36]. Current trends of patients undergoing SAVR show an increase in aortic root enlargement use from 3.9% to 6.3% in the United States [47]. Importantly, for younger low-risk patients, SAVR with concomitant root enlargement as the initial operation can facilitate implantation of a larger THV during a future valve-in-valve TAVR when the index bioprosthesis fails [33]. In select younger patients with a small aortic annulus and low bleeding risk, SAVR with a mechanical valve may be an appropriate strategy, as it avoids the need for subsequent reintervention and further minimizes the long-term risk of PPM.

### 3.4. Bicuspid Aortic Valve

Bicuspid aortic valve (BAV) is the most common congenital cardiac anomaly, affecting approximately 1–2% of the general population and accounting for nearly half of all cases of severe aortic stenosis in patients under 70 years of age [48]. Patients with BAV have been excluded from almost all landmark randomized controlled trials comparing TAVI with SAVR to date. Contemporary guidelines from both ACC/AHA and ESC/EACTS therefore continue to recommend SAVR as the preferred initial therapy for many BAV patients, especially those who are younger, have root or ascending aortic dilation, or are otherwise suitable for concomitant aortic surgery [2,49]. Observational registries demonstrate that in carefully selected older BAV patients with favorable CT anatomy, TAVR can achieve early outcomes comparable to those seen in tricuspid AS or SAVR, although with higher rates of paravalvular leak and stroke [50]. These findings may be attributable to the more elliptical annular geometry typical of BAV, extensive raphe or LVOT calcifications, and the increased need for pre- and post-dilation as well as THV repositioning [51,52]. Early data on low-risk patients, including the PARTNER 3 Bicuspid Registry, similarly suggest that BE THVs can perform well in anatomically favorable BAV phenotypes (without extensive raphe or subannular calcification), but long-term durability and coronary access considerations remain unresolved [53,54,55]. A meta-analysis including patients with BAV undergoing TAVR across all surgical-risk strata found that overall device success and 1-year mortality were comparable to outcomes in tricuspid aortic valve patients. However, higher rates of paravalvular leak, annular rupture, and cerebrovascular ischemic events in the BAV cohort were reported. These risks were particularly pronounced with SE THV [56].

In summary, the decision between SAVR and TAVR should be made through comprehensive Heart Team evaluation, with consideration of the current ACC/AHA and ESC guidelines favoring SAVR as the preferred intervention for most BAV patients—especially those who are younger or have concomitant aortic pathology [2,49]. Although further evidence is needed to clarify the optimal transcatheter strategy in this population, when TAVR is selected, the use of balloon-expandable valves may offer an advantage by reducing the incidence of paravalvular leak. These considerations, however, should emphasize the importance of individualized, anatomy-driven decision making within a lifetime management framework.

### 3.5. Paravalvular Leak

Paravalvular leak (PVL) is the main cause of post-procedural aortic regurgitation and occurs when there is incomplete apposition between a prosthetic valve and the native aortic annulus. PVL could cause important consequences on left ventricular volume load, promote adverse remodeling, and lead to heart failure. Furthermore, significant PVL could result in hemolysis requiring multiple transfusions and impaired long-term valve performance [57]. Multiple large studies and meta-analyses have shown that moderate or severe PVL is associated with a two- to three-fold increase in mortality [58,59], and growing evidence suggests that even mild PVL is linked to worse long-term outcomes compared with no or trace leak [60].

PVL after SAVR is uncommon because surgery allows direct excision of diseased leaflets and complete removal of annular and LVOT calcification, enabling a well-seated prosthesis with circumferential apposition. In contrast, TAVR relies on anchoring a prosthetic frame within a calcified, often irregular annulus, making it inherently more susceptible to residual PVL. Consistent with this, low-risk randomized trials have shown that moderate-to-severe PVL occurs significantly more frequently after TAVR (up to 3.4%) compared with SAVR (0.6%), and observed more often with SE THV than BE THV [61,62].

Although contemporary transcatheter heart valves incorporate enhanced sealing skirts, adaptive frame geometries, and greater radial force to reduce paravalvular leak (PVL) [63], residual regurgitation is still strongly influenced by a combination of anatomical and procedural factors. Key anatomic contributors include heavy or asymmetric annular or LVOT calcification, elliptical annular geometry and bicuspid valve morphology, and large or borderline annular dimensions that limit full circular expansion [64]. Procedural contributors for PVL include prosthesis–annulus size mismatch, inadequate oversizing, suboptimal implantation depth, malalignment, and incomplete expansion due to rigid calcific nodules [63]. Accordingly, comprehensive pre-procedural CT imaging is critical for assessing calcium burden, annular geometry, and sizing parameters, and for guiding an optimized deployment strategy that reduces the likelihood of PVL. As TAVR continues to expand into low-risk patients, SAVR should be considered in patients with heavy, asymmetric calcification patterns not only because of its higher risk of significant PVL, valve under-expansion, annular rupture and stroke but also to facilitate re-do procedures down the road [65].

### 3.6. Conduction Disturbances and Permanent Pacemaker Requirement

The His–Purkinje system runs in close proximity to the membranous septum and LVOT; thus deep or heavily calcified THV implantation can compress the bundle branch region and precipitate new left bundle branch block (LBBB) or high-grade AV block. Observational data and randomized trials consistently show higher rates of new permanent pacemaker implantation (PPI) after TAVR than after SAVR, particularly with self-expanding devices. Pooled analyses and registry data report PPI rates of 17–25% with self-expanding valves versus ~5–10% with balloon-expandable valves and 3–8% after SAVR [66,67]. In the low-risk PARTNER 3 trial (balloon-expandable TAVR vs. SAVR), new PPI was still more frequent after TAVR (6.5% vs. 4.0%), while the Evolut Low Risk trial (self-expanding vs. SAVR) showed an even larger gap (17.4% vs. 6.1%) [61,62]. Conduction abnormalities are not benign. A new LBBB and post-TAVR PPI have been associated with higher rates of heart failure hospitalization, less LV reverse remodeling, and increased mortality in several cohorts [68]. In patients with a longer life expectancy, right ventricular pacing and consequent ventricular desynchrony can contribute to a decline in LV function and overall cardiac performance over time [69]. Anatomical and procedural predictors of PPI include pre-existing right bundle branch block, prolonged PR interval, short membranous septum, heavy LVOT/annular calcification, narrow LVOT geometry, deeper implantation, and oversizing, which can be assessed and mitigated with meticulous CT-guided planning and “high” implantation strategies [70,71]. The risk of PPI should be carefully considered in the choice between TAVR and SAVR, especially in young patients. If TAVR is pursued, device characteristics and procedural techniques aimed at minimizing conduction injury is essential.

### 3.7. Concomitant Pathology

According to contemporary guidelines, SAVR remains the preferred strategy for patients with multivessel coronary artery disease (CAD) with high SYNTAX scores or significant left main CAD, bicuspid aortic valves and concomitant aortopathy ≥ 4.5 cm, or significant concomitant valvular pathology such as severe primary mitral regurgitation [2,3,49]. In younger patients with aortic stenosis where long-term durability, preservation of coronary access, and the cumulative impact of future reinterventions are critical, a Heart Team-based multidisciplinary evaluation is essential, particularly in cases with additional pathology that falls below formal surgical thresholds yet may influence procedural planning.

### 3.8. Transfemoral Access Feasibility

The majority of TAVR procedures are performed via transfemoral (TF) access, which remains the safest and most physiologic route for valve delivery. In trials such as Evolut Low Risk, alternative access was required in only ~1% of cases, while inability to undergo TF access was an explicit exclusion criterion in PARTNER 3 [61,62]. Alternative access options—including transthoracic approaches (transapical and direct aortic) and non-femoral peripheral approaches (transaxillary/subclavian, transcarotid, and transcaval)—are typically reserved for patients with prohibitive iliofemoral anatomy [72]. These patients more often have peripheral arterial disease, severe iliofemoral calcification, obesity, or hostile vascular anatomy. Across multiple registries and meta-analyses, all alternative-access TAVR routes carry higher rates of mortality, stroke, bleeding, and vascular complications compared with TF-TAVR, with transapical and direct aortic access demonstrating the poorest outcomes [73,74]. Emerging technologies such as intravascular lithotripsy offer a promising strategy to facilitate TF access in patients with heavily calcified iliofemoral vessels, but long-term data and randomized evidence remain limited [75]. Given the clearly inferior outcomes associated with alternative-access TAVR, and the absence of these patients from pivotal low-risk trials, SAVR remains the preferred treatment strategy for low-surgical-risk patients in whom TF access is not feasible.

## 4. Lifetime Management Strategies: Reintervention

Reintervention rates for bioprosthetic valves are reported to be approximately 7% at 10 years and 15% at 20 years, though these rates vary substantially based on patient age as well as the design, generation, and specific model of the implanted bioprosthesis [11]. According to the Valve Academic Research Consortium-3 (VARC-3) definitions, bioprosthetic aortic valve failure may occur via four principal mechanisms: structural valve deterioration (SVD), non-structural valve deterioration (non-SVD), valve thrombosis, and infective endocarditis [76]. When a THV fails, reintervention options include redo TAVR (TAV-in-TAV) or surgical explant of the THV followed by SAVR, each with distinct anatomic feasibility thresholds, risks, and durability considerations. In patients that initially underwent SAVR, options for bioprosthetic failure are TAV-in-SAV or redo SAVR.

### 4.1. When TAVR Fails: Redo TAVR

**Figure 5:**
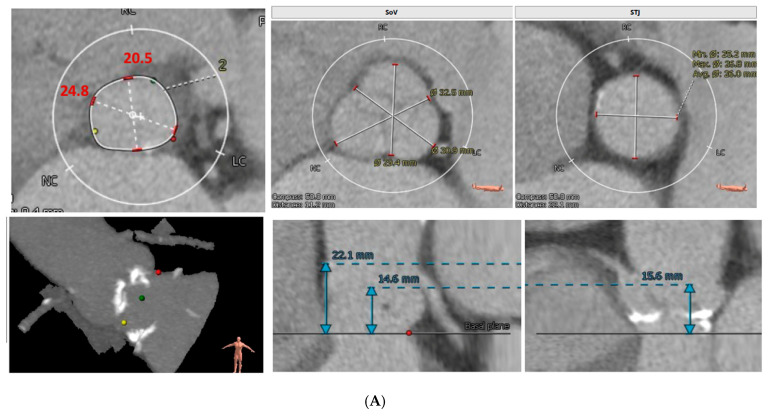

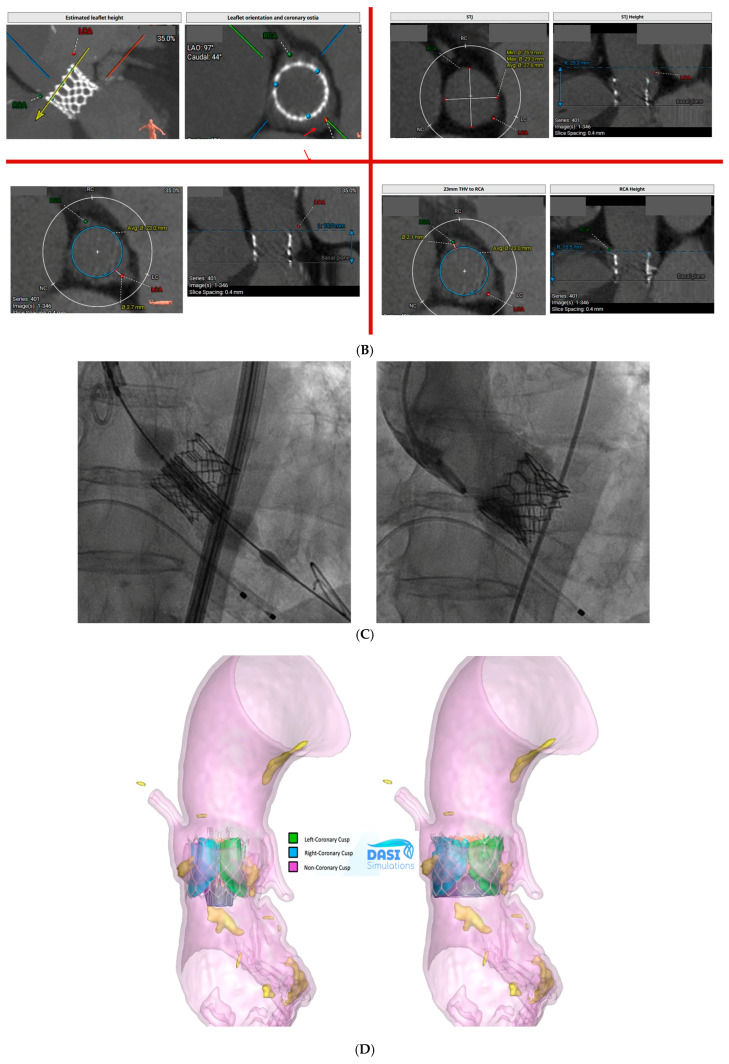

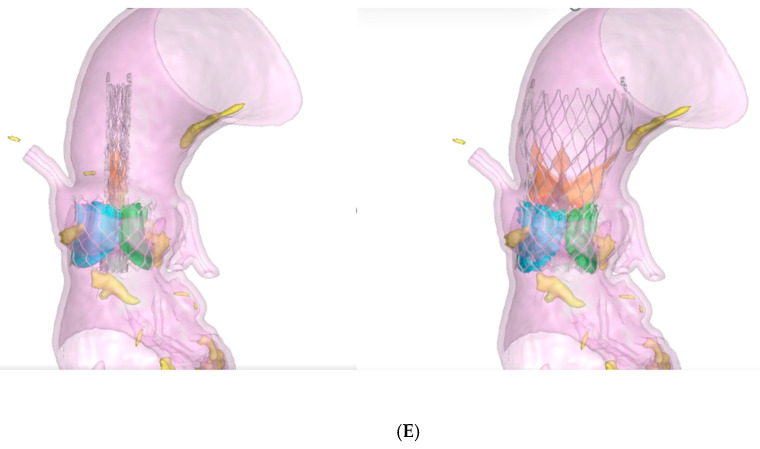
Favorable Case for Redo TAVR. (**A**) Index TAVR Procedure. Favorable TAVR anatomy for index procedure, characterized by suitable annular dimensions, adequate sinus of Valsalva and STJ size, safe coronary heights, absence of high-risk calcification, and supportive 3D sinus geometry. (**B**) TAVR with structural valve deterioration. Several years later, CT imaging demonstrates structural valve deterioration. Elevated gradients also seen by echocardiography and patient noted symptomatic. Redo TAVR is favorable given adequate sinus and STJ dimensions, safe coronary heights, and sufficient valve-to-coronary clearance. (**C**) Fluoroscopic Imaging of TAVR-in-TAVR Implantation. Sequential fluoroscopic images of a patient who had a TAVR valve 7 years prior with structural valve deterioration. This illustrates positioning and deployment of the new transcatheter valve within the prior TAVR valve prosthesis, demonstrating stable coaxial alignment, and adequate frame expansion within the existing valve structure. (**D**) DASI (Dynamic Aortic Sinus Interaction) simulation of BE TAVR 23 in BE TAVR 23 Expansion showing favorable TAVR in TAVR. Panel (**E**): DASI simulation of SE TAVR 26 in BE TAVR 23. Expansion is favorable. (**A**–**C**) are examples of CT images from authors institution. Panel (**D**) is from commercial DASI simulation with permission to use as it is showing a commercial TAVR valve (discussed with DASI).

**Figure 6:**
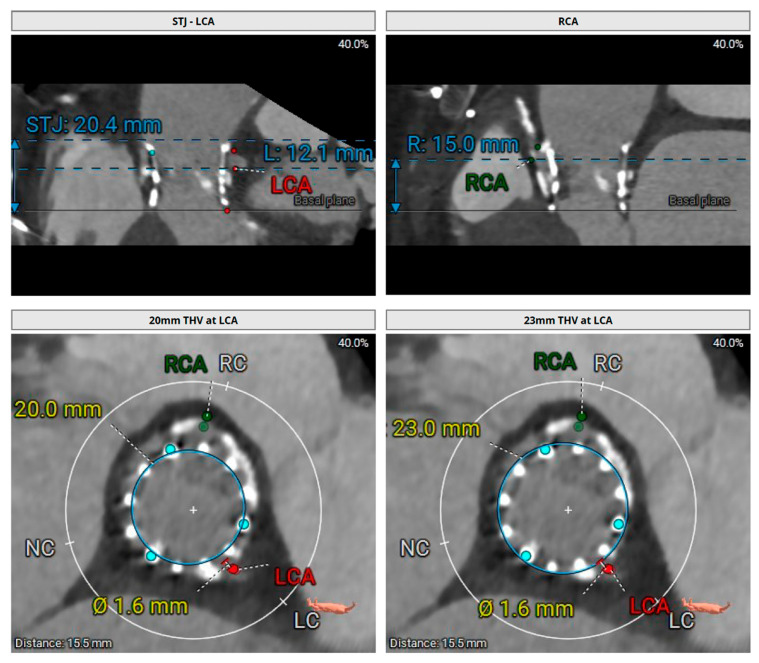
An Unfavorable Case for Re-do TAVR. Pre-procedure CT scan of TAVR valve deterioration showing high-risk anatomy noted by the sinus sequestration which occurs with small valve-to aorta clearance at the STJ (<2 mm) which can jeopardize re-access. Example of TAVR CT images from authors’ institution.

Early experience with TAV-in-TAV demonstrated favorable short-term outcomes, with very low reported rates of valve embolization, coronary obstruction, or emergent conversion to open surgery in appropriately selected patients [33]. However, not all TAVR patients may be eligible, because of unfavorable anatomy or other clinical indications. Anatomic considerations may include obstructed or low coronary ostia, small annulus, supra-annular index THV, previous redo TAVR, or anticipated mitral valve impingement [77]. Data from a multicenter, international registry (EXPLANT-TAVR) that retrospectively reviewed patients that underwent TAVR explanation reported that redo TAVR was not feasible in 34% of their cohort because of unfavorable anatomy (26.8%) and unsatisfactory results after a valve-in-valve procedure (7.2%) [77]. Careful multidetector CT (MDCT) assessment to evaluate the risks for coronary obstruction, sinus sequestration, severe PPM, and suboptimal hemodynamic status is needed to determine if redo TAVR would be appropriate. Two key principles guide feasibility evaluation: (1) when the coronary ostial ‘risk plane’ lies above the neoskirt created by the index transcatheter valve, there is no risk of coronary obstruction, whereas (2) if the neoskirt height exceeds the coronary ostia or the STJ, there is potential for coronary obstruction or complete sinus sequestration during a second valve deployment [35]. MDCT-derived thresholds further refine risk stratification: a virtual valve-to-coronary distance < 4 mm predicts high risk of coronary obstruction, while a valve-to-aorta distance < 2 mm at the STJ is strongly associated with sinus sequestration and compromised sinus egress [78]. Additional factors (including leaflet length of the index THV, frame height, commissural orientation, and the geometry of the sinus of Valsalva) also influence the ability of displaced leaflets to clear the coronary ostia (Figure 5 and Figure 6). Because commissural misalignment can position THV posts directly in front of the coronaries and elevate neoskirt height, achieving commissural alignment at the index procedure is critical to preserving redo TAVR feasibility and future coronary access.

In the context of TAV-in-TAV, options for coronary protection can be limited (Figure 4). The BASILICA technique is significantly limited because the rigid THV frame restricts leaflet splay, reducing the technique’s ability to create an adequate flow channel to the coronaries. Chimney stenting has concerns for long-term stent patency given that the coronary stent becomes trapped between two layers of metallic valve frame, potentially exposed to turbulent flow, neointimal hyperplasia, and mechanical fatigue [78]. A more recent modification, balloon-assisted BASILICA, which pre-dilates and separates the leaflets before electrosurgical laceration, has shown early promise in expanding the leaflet splay zone and improving procedural success. However, this approach still requires specialized electrocautery equipment, advanced operator expertise, and further clinical validation before it can be widely adopted [79].

The need for concomitant cardiac surgery is an important exclusion criterion for redo TAVR. Additionally, hemodynamic complications like PVL and PPM are inherently difficult to correct with a second transcatheter valve, which may necessitate TAVR explant and SAVR [80,81]. Furthermore, prosthetic valve endocarditis, which occurs in approximately 5–10% of bioprosthetic valve failures, requires complete removal of infected material and therefore contraindicates redo TAVR [81].

### 4.2. When TAVR Fails: TAVR Explant and SAVR

Following the publication of the ACC/AHA low-risk guidelines in 2019 and the consequent rise in TAVR utilization in lower risk patients, an STS Database study reported an exponential uptick in TAVR explant volume. The study found the annual number of patients undergoing SAVR following TAVR explant increased from 14 in 2012 to 828 in 2023, a nearly 1.5-fold growth rate per year. Currently, TAVR explantation is the fastest-growing cardiac surgical procedure in the United States, and increasing surgeon experience is expected to improve outcomes [82].

Explantation of THVs is a high-risk operation characterized by several technical challenges. As TAVR prostheses age, progressive neoendothelialization and fibrotic ingrowth often bind the transcatheter valve frame to surrounding aortic structures. In many cases, particularly when the THV has been implanted for more than a year, explant requires extensive aortic endarterectomy, and not infrequently aortic root or ascending aortic replacement, depending on where the frame has fused. Patterns of endothelialization differ by device type: balloon-expandable valves tend to ingrow within the aortic root, whereas self-expanding valves more commonly endothelialize at the sinotubular junction, sometimes necessitating ascending aortic replacement during explant. These anatomical complexities help explain why TAVR explant has increased operative mortality.

Additionally, patients referred for explant of a failed TAVR prosthesis frequently represent a high-risk cohort due to prior ineligibility for surgery, significant comorbidities, frailty, challenging anatomy, complex structural issues, previous operations, heavy aortic calcification, or a small aortic annulus. In the registry data of TAVR explants, in-hospital mortality was 12%, and 1-year mortality was 29%. Given the rarity of TAVR explantation (<1% of TAVR procedures), even high-volume TAVR centers perform very few annually, resulting in significant variations in mortality and morbidity [77]. Emerging evidence, including findings from Fukuhara et al., demonstrates that even patients who were initially classified as low surgical risk may experience disproportionately elevated observed-to-expected mortality when undergoing TAVR explantation, highlighting the inadequacy of traditional risk models for this unique population [83]. The Society of Thoracic Surgeons has introduced updated Adult Cardiac Surgery Risk Calculators that now integrate advanced assessments for surgical aortic valve replacement and account for patients with prior TAVR procedures. These enhancements to the STS risk score improve the precision of risk prediction for this expanding, high-risk patient group [84].

Redo TAVR is not suitable for all patients. Those with prohibitive coronary anatomy, multivalvular involvement, severe patient–prosthetic mismatch, or endocarditis should be referred for TAVR explant. Data from the EXPLANT-TAVR Registry reported that the main indication for TAVR explant was endocarditis (43%) and THV dysfunction (SVD 20%, PVL 18% and PPM 11%) [77]. In this treatment strategy, the second valve intervention provides the durability of a *new* surgical aortic valve replacement (SAVR). Given that a newly implanted SAVR typically lasts 8–15 years, patients can anticipate roughly another decade of valve function before requiring a third procedure. This approach is considered theoretically advantageous, as it increases the likelihood that patients will need only one open-heart surgery overall, with the third intervention, if needed, most likely being a less invasive TAVR-in-SAV procedure.

### 4.3. When SAVR Fails: Redo SAVR

Prior cardiac surgery is a significant risk factor for undergoing SAVR again, primarily because scar tissue and adhesions from the initial operation increase procedural complexity. Redo SAVR is associated with 4.7% in-hospital mortality, and when compared with initial SAVR, redo SAVR had higher mortality and morbidity and longer hospital length of stay [85]. Moreover, short-term outcomes following redo SAVR tend to be less favorable than those seen with TAVR-in-SAV [86]. Complications such as bleeding requiring transfusion, acute kidney injury requiring dialysis, new pacemaker implantation, and heart failure readmissions were higher after redo SAVR. However, a study utilizing a large administrative French database suggests that, upon longer-term follow-up, major cardiovascular outcomes eventually converge and become comparable [87]. In a registry-based study, Onorati et al. found that approximately two-thirds of patients had freedom from re-intervention at 10 years [88]. Benefits of redo SAVR over TAV-in-SAV include the opportunity for enlarging the aortic annulus for implantation of a large valve, that it reduces risk of coronary obstruction and facilitates coronary access, lower postoperative gradients, less PVL and leaflet thrombosis. Importantly, redo SAVR offers the possibility of a future TAV-in-SAV as the third intervention, which is an attractive option as patients age and become less suitable candidates for another open-heart procedure [78].

### 4.4. When SAVR Fails: Valve-in-Valve TAVR

Patients undergoing TAVR-in-SAVR generally experience fewer mechanical complications (such as annular rupture or new conduction abnormalities) because the existing surgical bioprosthesis serves as a stabilizing scaffold for valve deployment [33]. However, valve-in-valve TAVR is associated with a higher risk of persistently elevated postoperative gradients, coronary obstruction, PPM, valve thrombosis, and malposition of the transcatheter valve. Emerging evidence indicates that long-term survival following TAVR-in-SAVR is strongly related to postoperative hemodynamics, which are largely determined by the characteristics of the underlying surgical valve. In particular, small stented surgical valves with internal diameters under 20 mm are associated with a markedly increased risk of residual stenosis after the procedure [89]. In this context, it is crucial to implant the largest feasible surgical bioprosthesis, along with aortic root enlargement when appropriate, during the index SAVR to maximize the effective orifice area and ensure more favorable hemodynamics during a future TAVR-in-SAVR procedure. Furthermore, if TAV-in-SAV fails, the possibility of another valve-in-valve therapy (TAVR-in-TAVR-in-SAVR) may exist, specifically in patients with larger aortic roots.

Patient–prosthesis mismatch (PPM) and the potential for coronary obstruction are key factors that must be evaluated when planning a valve-in-valve TAVR procedure. Techniques such as bioprosthetic valve fracture (BVF) and bioprosthetic valve remodeling (BVR) can enhance procedural outcomes, in selected patients, by allowing greater expansion of the transcatheter valve, thereby lowering transvalvular gradients and increasing the effective orifice area, benefits that are particularly important in patients with smaller surgical valves. These methods involve high-pressure inflation of a noncompliant balloon to fracture or stretch the surgical valve ring, either before or after TAVR deployment. However, certain surgical bioprostheses, including the Medtronic Hancock II and Medtronic Avalus valves, are not amenable to stretching or fracturing [90]. To mitigate the risk of coronary obstruction, preventive techniques such as chimney stenting and electrosurgical leaflet modification may be used [91]. Nevertheless, in younger or low-risk patients who demonstrate high predicted coronary obstruction risk with valve-in-valve TAVR, redo SAVR remains the preferred strategy to ensure long-term safety and durability [35].

### 4.5. Lifetime Management Strategies: AI-Guided Simulation

The traditional method of THV selection relies on cardiac-gated CT measurements of the aortic annulus and root; however this anatomical approach cannot fully predict how a chosen valve will interact with the patient’s native structures during or after TAVI deployment. Computer-based and AI-enhanced simulations can model the dynamic behavior of different THV types and sizes within a patient’s specific anatomy, providing insight into risks such as annular rupture, conduction disturbances, and paravalvular leak. These simulations depend on deep-learning algorithms capable of integrating the geometric and mechanical properties of the aortic root with the stress–strain characteristics of various valve designs. Simulation models offer a powerful adjunct to standard CT-based assessment by providing patient-specific, AI-enhanced computational simulations that help not only guide THV selection in cases with complex anatomic features but also help depict lifetime management of young TAVR patients by simulating subsequent THV implantations and provide insight into coronary access and residual valve areas [92]. FEops HEARTguide™ (FEops NV, Ghent, Belgium) and DASI are commercially available simulation models, among others, that have shown promise in optimizing outcomes and should be considered as an additional tool in lifetime management of aortic stenosis [93,94].

## 5. Conclusions

**Figure 7:**
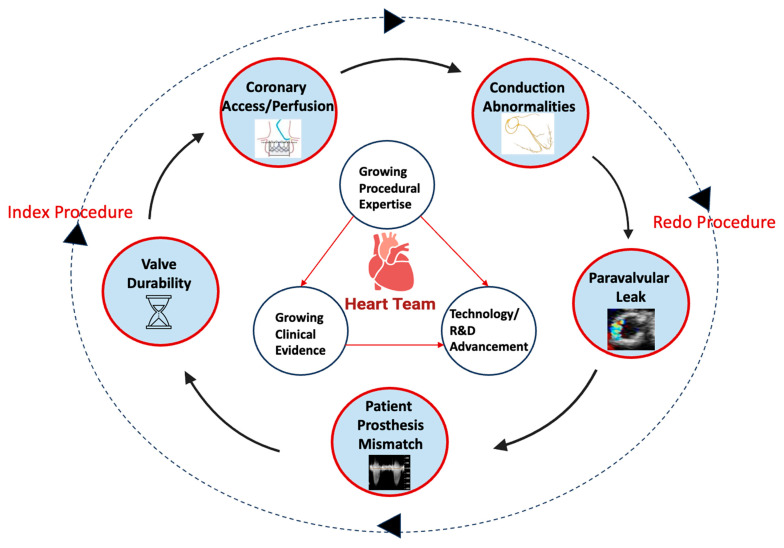
Key Determinants Influencing Lifetime Aortic Valve Management. This diagram highlights the interplay of growing clinical expertise, ongoing technological innovation, and expanding clinical evidence that guides the modern management of aortic stenosis. Important challenges remain throughout the lifespan of a bioprosthetic valve. These include structural valve degeneration, limitations in coronary access, pacemaker requirement, paravalvular leak, and prosthesis–patient mismatch. These challenges may accumulate over time and ultimately necessitate repeat intervention. Accordingly, thoughtful planning of the initial valve procedure is essential, with careful attention to anatomical feasibility, valve type, and future reintervention options. Aortic valve replacement should be approached as a lifetime strategy rather than a single procedural event, anticipating the possibility of sequential therapies as patients live longer with valve disease. Original figure created by the authors.

Lifetime management of aortic stenosis requires a nuanced, future-oriented approach that extends beyond the immediate success of the index procedure. Careful selection between SAVR and TAVR at the initial intervention, guided by patient age, anatomy, comorbidities, and long-term goals, sets the foundation for future treatment pathways (Figure 7). As bioprosthetic valves inevitably deteriorate, clinicians must be prepared to tailor reintervention strategies based on anatomical constraints, procedural risks, and patient-related factors. Considerations such as patient–prosthesis mismatch, coronary obstruction risk, and the feasibility of future transcatheter therapies are central to optimizing outcomes across a patient’s lifetime. By integrating these principles and anticipating the sequence of interventions, Heart Teams can deliver personalized, durable, and safe management for patients with aortic stenosis across the full course of their disease.

## Data Availability

The original contributions presented in this study are included in the article. Further inquiries can be directed to the corresponding author.

## References

[1] Whelton S.P., Jha K., Dardari Z., Razavi A.C., Boakye E., Dzaye O., Verghese D., Shah S., Budoff M.J., Matsushita K. (2024). Prevalence of Aortic Valve Calcium and the Long-Term Risk of Incident Severe Aortic Stenosis. JACC Cardiovasc. Imaging.

[2] Otto C.M., Nishimura R.A., Bonow R.O., Carabello B.A., Erwin J.P., Gentile F., Jneid H., Krieger E.V., Mack M., McLeod C. (2021). 2020 ACC/AHA Guideline for the Management of Patients with Valvular Heart Disease: A Report of the American College of Cardiology/American Heart Association Joint Committee on Clinical Practice Guidelines. Circulation.

[3] Praz F., Borger M.A., Lanz J., Marin-Cuartas M., Abreu A., Adamo M., Ajmone Marsan N., Barili F., Bonaros N., Cosyns B. (2025). 2025 ESC/EACTS Guidelines for the management of valvular heart disease: Developed by the task force for the management of valvular heart disease of the European Society of Cardiology (ESC) and the European Association for Cardio-Thoracic Surgery (EACTS). Eur. Heart J..

[4] Mack M.J., Leon M.B., Thourani V.H., Pibarot P., Hahn R.T., Genereux P., Kodali S.K., Kapadia S.R., Cohen D.J., Pocock S.J. (2023). Transcatheter Aortic-Valve Replacement in Low-Risk Patients at Five Years. N. Engl. J. Med..

[5] Leon M.B., Mack M.J., Pibarot P., Hahn R.T., Thourani V.H., Kodali S.H., Généreux P., Kapadia S.R., Cohen D.J., Pocock S.J. (2025). Transcatheter or Surgical Aortic-Valve Replacement in Low-Risk Patients at 7 Years. N. Engl. J. Med..

[6] Thyregod H.G.H., Jørgensen T.H., Ihlemann N., Steinbrüchel D.A., Nissen H., Kjeldsen B.J., Petursson P., De Backer O., Olsen P.S., Søndergaard L. (2024). Transcatheter or surgical aortic valve implantation: 10-year outcomes of the NOTION trial. Eur. Heart J..

[7] Sharma T., Krishnan A.M., Lahoud R., Polomsky M., Dauerman H.L. (2022). National Trends in TAVR and SAVR for Patients With Severe Isolated Aortic Stenosis. JACC.

[8] Carroll J.D., Mack M.J., Vemulapalli S., Herrmann H.C., Gleason T.G., Hanzel G., Deeb G.M., Thourani V.H., Cohen D.J., Desai N. (2020). STS-ACC TVT Registry of Transcatheter Aortic Valve Replacement. JACC.

[9] Ryan W.H., Squiers J.J., Harrington K.B., Goodenow T., Rawitscher C., Schaffer J.M., DiMaio J.M., Brinkman W.T. (2021). Long-term outcomes of the Ross procedure in adults. Ann. Cardiothorac. Surg..

[10] El-Hamamsy I., Toyoda N., Itagaki S., Stelzer P., Varghese R., Williams E.E., Erogova N., Adams D.H. (2022). Propensity-Matched Comparison of the Ross Procedure and Prosthetic Aortic Valve Replacement in Adults. J. Am. Coll. Cardiol..

[11] Saadi R.P., Tagliari A.P., Zaid S., Tang G.H.L. (2024). Considerations for lifetime management of aortic stenosis: Transcatheter aortic valve replacement, surgical aortic valve replacement, and timing of therapy. JTCVS Struct. Endovasc..

[12] Rowse P.G., Schaff H.V. (2022). Controversy pro: Mechanical AVR for better long-term survival of 50–70 years old. Prog. Cardiovasc. Dis..

[13] Mazine A., El-Hamamsy I., Verma S., Peterson M.D., Bonow R.O., Yacoub M.H., David T.E., Bhatt D.L. (2018). Ross Procedure in Adults for Cardiologists and Cardiac Surgeons: JACC State-of-the-Art Review. J. Am. Coll. Cardiol..

[14] Brennan J.M., Edwards F.H., Zhao Y., O’Brien S.M., Douglas P.S., Peterson E.D. (2012). Long-Term Survival After Aortic Valve Replacement Among High-Risk Elderly Patients in the United States. Circulation.

[15] Forrest J.K., Yakubov S.J., Deeb G.M., Gada H., Mumtaz M.A., Ramlawi B., Bajwa T., Crouch J., Merhi W., Sang S.L.W. (2025). 5-Year Outcomes After Transcatheter or Surgical Aortic Valve Replacement in Low-Risk Patients with Aortic Stenosis. JACC.

[16] Ribeiro H.B., Rodés-Cabau J., Blanke P., Leipsic J., Kwan Park J., Bapat V., Makkar R., Simonato M., Barbanti M., Schofer J. (2018). Incidence, predictors, and clinical outcomes of coronary obstruction following transcatheter aortic valve replacement for degenerative bioprosthetic surgical valves: Insights from the VIVID registry. Eur. Heart J..

[17] Ibrahim H., Chaus A., Alkhalil A., Prescher L., Kleiman N. (2024). Coronary Artery Obstruction After Transcatheter Aortic Valve Implantation: Past, Present, and Future. Circ. Cardiovasc. Interv..

[18] Ribeiro H.B., Webb J.G., Makkar R.R., Cohen M.G., Kapadia S.R., Kodali S., Tamburino C., Barbanti M., Chakravarty T., Jilaihawi H. (2013). Predictive Factors, Management, and Clinical Outcomes of Coronary Obstruction Following Transcatheter Aortic Valve Implantation: Insights From a Large Multicenter Registry. J. Am. Coll. Cardiol..

[19] He A., Wilkins B., Lan N.S.R., Othman F., Sehly A., Bhat V., Jaltotage B., Dwivedi G., Leipsic J., Ihdayhid A.R. (2024). Cardiac computed tomography post-transcatheter aortic valve replacement. J. Cardiovasc. Comput. Tomogr..

[20] Tarantini G., Nai Fovino L., Scotti A., Massussi M., Cardaioli F., Rodinò G., Benedetti A., Boiago M., Matsuda Y., Continisio S. (2022). Coronary Access After Transcatheter Aortic Valve Replacement with Commissural Alignment: The ALIGN-ACCESS Study. Circ. Cardiovasc. Interv..

[21] Ochiai T., Oakley L., Sekhon N., Komatsu I., Flint N., Kaewkes D., Yoon S.-H., Raschpichler M., Patel V., Tiwana R. (2020). Risk of Coronary Obstruction Due to Sinus Sequestration in Redo Transcatheter Aortic Valve Replacement. JACC Cardiovasc. Interv..

[22] Bapat V. (2014). Valve-in-valve apps: Why and how they were developed and how to use them. Eurointerv. J. Eur. Collab. Work. Group Interv. Cardiol. Eur. Soc. Cardiol..

[23] Grubb K.J., Ueyama H.A., Tom S.K., Reul R.M., Nissen A.P., Tully A., Camaj A., Lisko J., Xie J., Norton E.L. (2025). How to avoid transcatheter aortic valve replacement explant as the second valve procedure: Image assessment for the index transcatheter aortic valve replacement. Ann. Cardiothorac. Surg..

[24] Chen S., Pop A., Prasad Dasi L., George I. (2025). Lifetime Management for Aortic Stenosis: Strategy and Decision-Making in the Current Era. Ann. Thorac. Surg..

[25] Khan J.M., Greenbaum A.B., Babaliaros V.C., Rogers T., Eng M.H., Paone G., Leshnower B.G., Reisman M., Satler L., Waksman R. (2019). The BASILICA Trial: Prospective Multicenter Investigation of Intentional Leaflet Laceration to Prevent TAVR Coronary Obstruction. JACC Cardiovasc. Interv..

[26] Mercanti F., Rosseel L., Neylon A., Bagur R., Sinning J.-M., Nickenig G., Grube E., Hildick-Smith D., Tavano D., Wolf A. (2020). Chimney Stenting for Coronary Occlusion During TAVR: Insights from the Chimney Registry. JACC Cardiovasc. Interv..

[27] Tchétché D., Kodali S.K., Dvir D. (2022). First dedicated transcatheter leaflet splitting device: The ShortCut device. EuroIntervention.

[28] Rogers T., Bruce C.G. (2023). Orthotopic stenting after BASILICA—An indicator of procedural success. EuroIntervention.

[29] Zebhi B., Lazkani M., Bark D. (2021). Calcific Aortic Stenosis—A Review on Acquired Mechanisms of the Disease and Treatments. Front. Cardiovasc. Med..

[30] Khodaee F., Qiu D., Dvir D., Azadani A.N. (2019). Reducing the risk of leaflet thrombosis in transcatheter aortic valve-in-valve implantation by BASILICA: A computational simulation study. EuroIntervention.

[31] Krishnaswamy A., Kapadia S.R., Suri R. (2020). Avoiding danger—Addressing the specter of coronary obstruction during transcatheter aortic valve replacement. J. Thorac. Cardiovasc. Surg..

[32] Bahlmann E., Cramariuc D., Minners J., Lønnebakken M.T., Ray S., Gohlke-Baerwolf C., Nienaber C.A., Jander N., Seifert R., Chambers J.B. (2017). Small aortic root in aortic valve stenosis: Clinical characteristics and prognostic implications. Eur. Heart J. Cardiovasc. Imaging.

[33] Russo G., Tang G.H.L., Sangiorgi G., Pedicino D., Enriquez-Sarano M., Maisano F., Taramasso M. (2022). Lifetime Management of Aortic Stenosis: Transcatheter Versus Surgical Treatment for Young and Low-Risk Patients. Circ. Cardiovasc. Interv..

[34] Freitas-Ferraz A.B., Tirado-Conte G., Dagenais F., Ruel M., Al-Atassi T., Dumont E., Mohammadi S., Bernier M., Pibarot P., Rodés-Cabau J. (2019). Aortic Stenosis and Small Aortic Annulus. Circulation.

[35] Gupta T., Malaisrie S.C., Batchelor W., Boudoulas K.D., Davidson L., Ibebuogu U.N., Kpodonu J., Singh R., Sultan I., Theriot M. (2025). Review of the decision making approach to treating young and low-risk patients with aortic stenosis. J. Thorac. Cardiovasc. Surg..

[36] Herrmann H.C., Desai N.D. (2024). Incidence, Implications, and Treatment of Patients with Severe Aortic Stenosis and Small Aortic Annulus. Circulation.

[37] Deeb G.M., Chetcuti S.J., Yakubov S.J., Patel H.J., Grossman P.M., Kleiman N.S., Heiser J., Merhi W., Zorn G.L., Tadros P.N. (2018). Impact of Annular Size on Outcomes After Surgical or Transcatheter Aortic Valve Replacement. Ann. Thorac. Surg..

[38] Rodés-Cabau J., Pibarot P., Suri R.M., Kodali S., Thourani V.H., Szeto W.Y., Svensson L.G., Dumont E., Xu K., Hahn R.T. (2014). Impact of aortic annulus size on valve hemodynamics and clinical outcomes after transcatheter and surgical aortic valve replacement: Insights from the PARTNER Trial. Circ. Cardiovasc. Interv..

[39] Puri R., Byrne J., Muller R., Baumbach H., Eltchaninoff H., Redwood S., Cheema A., Dubois C., Ihlberg L., Wijeysundera H.C. (2017). Transcatheter aortic valve implantation in patients with small aortic annuli using a 20 mm balloon-expanding valve. Heart.

[40] Pasic M., Unbehaun A., Buz S., Drews T., Hetzer R. (2015). Annular rupture during transcatheter aortic valve replacement: Classification, pathophysiology, diagnostics, treatment approaches, and prevention. JACC Cardiovasc. Interv..

[41] Voigtländer L., Kim W.-K., Mauri V., Goßling A., Renker M., Sugiura A., Linder M., Schmidt T., Schofer N., Westermann D. (2021). Transcatheter aortic valve implantation in patients with a small aortic annulus: Performance of supra-, intra- and infra-annular transcatheter heart valves. Clin. Res. Cardiol..

[42] Szerlip M., Gualano S., Holper E., Squiers J.J., White J.M., Doshi D., Williams M.R., Hahn R.T., Webb J.G., Svensson L.G. (2018). Sex-Specific Outcomes of Transcatheter Aortic Valve Replacement with the SAPIEN 3 Valve: Insights from the PARTNER II S3 High-Risk and Intermediate-Risk Cohorts. JACC Cardiovasc. Interv..

[43] Mauri V., Kim W.K., Abumayyaleh M., Walther T., Moellmann H., Schaefer U., Conradi L., Hengstenberg C., Hilker M., Wahlers T. (2017). Short-Term Outcome and Hemodynamic Performance of Next-Generation Self-Expanding Versus Balloon-Expandable Transcatheter Aortic Valves in Patients with Small Aortic Annulus: A Multicenter Propensity-Matched Comparison. Circ. Cardiovasc. Interv..

[44] Herrmann H.C., Mehran R., Blackman D.J., Bailey S., Möllmann H., Abdel-Wahab M., Ben Ali W., Mahoney P.D., Ruge H., Wood D.A. (2024). Self-Expanding or Balloon-Expandable TAVR in Patients with a Small Aortic Annulus. N. Engl. J. Med..

[45] Pibarot P., Weissman N.J., Stewart W.J., Hahn R.T., Lindman B.R., McAndrew T., Kodali S.K., Mack M.J., Thourani V.H., Miller D.C. (2014). Incidence and sequelae of prosthesis-patient mismatch in transcatheter versus surgical valve replacement in high-risk patients with severe aortic stenosis: A PARTNER trial cohort—A analysis. J. Am. Coll. Cardiol..

[46] Thyregod H.G.H., Steinbrüchel D.A., Ihlemann N., Ngo T.A., Nissen H., Kjeldsen B.J., Chang Y., Hansen P.B., Olsen P.S., Søndergaard L. (2016). No clinical effect of prosthesis-patient mismatch after transcatheter versus surgical aortic valve replacement in intermediate- and low-risk patients with severe aortic valve stenosis at mid-term follow-up: An analysis from the NOTION trial. Eur. J. Cardio-Thorac. Surg. Off. J. Eur. Assoc. Cardio-Thorac. Surg..

[47] Hawkins R.B., Beller J.P., Mehaffey J.H., Charles E.J., Quader M.A., Rich J.B., Kiser A.C., Joseph M., Speir A.M., Kern J.A. (2019). Incremental Risk of Annular Enlargement: A Multi-Institutional Cohort Study. Ann. Thorac. Surg..

[48] Michelena H.I., Prakash S.K., Della Corte A., Bissell M.M., Anavekar N., Mathieu P., Bossé Y., Limongelli G., Bossone E., Benson D.W. (2014). Bicuspid Aortic Valve. Circulation.

[49] Vahanian A., Beyersdorf F., Praz F., Milojevic M., Baldus S., Bauersachs J., Capodanno D., Conradi L., De Bonis M., De Paulis R. (2022). 2021 ESC/EACTS Guidelines for the management of valvular heart disease. Eur. Heart J..

[50] Makkar R.R., Yoon S.-H., Leon M.B., Chakravarty T., Rinaldi M., Shah P.B., Skipper E.R., Thourani V.H., Babaliaros V., Cheng W. (2019). Association Between Transcatheter Aortic Valve Replacement for Bicuspid vs Tricuspid Aortic Stenosis and Mortality or Stroke. JAMA.

[51] Frangieh A.H., Kasel A.M. (2017). TAVI in Bicuspid Aortic Valves ‘Made Easy’. Eur. Heart J..

[52] Frangieh A.H., Michel J., Deutsch O., Joner M., Pellegrini C., Rheude T., Bleiziffer S., Kasel A.M. (2019). Aortic annulus sizing in stenotic bicommissural non-raphe-type bicuspid aortic valves: Reconstructing a three-dimensional structure using only two hinge points. Clin. Res. Cardiol..

[53] Williams M.R., Jilaihawi H., Makkar R., O’Neill W.W., Guyton R., Malaisrie S.C., Brown D.L., Blanke P., Leipsic J.A., Pibarot P. (2022). The PARTNER 3 Bicuspid Registry for Transcatheter Aortic Valve Replacement in Low-Surgical-Risk Patients. JACC Cardiovasc. Interv..

[54] Deeb G.M., Reardon M.J., Ramlawi B., Yakubov S.J., Chetcuti S.J., Kleiman N.S., Mangi A.A., Zahr F., Song H.K., Gada H. (2022). Propensity-Matched 1-Year Outcomes Following Transcatheter Aortic Valve Replacement in Low-Risk Bicuspid and Tricuspid Patients. JACC Cardiovasc. Interv..

[55] Michel J.M., Frangieh A.H., Giacoppo D., Alvarez-Covarrubias H.A., Pellegrini C., Rheude T., Deutsch O., Mayr N.P., Rumpf P.M., Stähli B.E. (2021). Safety and efficacy of minimalist transcatheter aortic valve implantation using a new-generation balloon-expandable transcatheter heart valve in bicuspid and tricuspid aortic valves. Clin. Res. Cardiol..

[56] Montalto C., Sticchi A., Crimi G., Laricchia A., Khokhar A.A., Giannini F., Reimers B., Colombo A., Latib A., Waksman R. (2021). Outcomes After Transcatheter Aortic Valve Replacement in Bicuspid Versus Tricuspid Anatomy: A Systematic Review and Meta-Analysis. JACC Cardiovasc. Interv..

[57] Généreux P., Head S.J., Hahn R., Daneault B., Kodali S., Williams M.R., van Mieghem N.M., Alu M.C., Serruys P.W., Kappetein A.P. (2013). Paravalvular Leak After Transcatheter Aortic Valve Replacement. JACC.

[58] Athappan G., Patvardhan E., Tuzcu E.M., Svensson L.G., Lemos P.A., Fraccaro C., Tarantini G., Sinning J.-M., Nickenig G., Capodanno D. (2013). Incidence, predictors, and outcomes of aortic regurgitation after transcatheter aortic valve replacement: Meta-analysis and systematic review of literature. J. Am. Coll. Cardiol..

[59] Takagi H., Umemoto T. (2016). Impact of paravalvular aortic regurgitation after transcatheter aortic valve implantation on survival. Int. J. Cardiol..

[60] Okuno T., Tomii D., Heg D., Lanz J., Praz F., Stortecky S., Reineke D., Windecker S., Pilgrim T. (2022). Five-year outcomes of mild paravalvular regurgitation after transcatheter aortic valve implantation. EuroIntervention.

[61] Mack M.J., Leon M.B., Thourani V.H., Makkar R., Kodali S.K., Russo M., Kapadia S.R., Malaisrie S.C., Cohen D.J., Pibarot P. (2019). Transcatheter Aortic-Valve Replacement with a Balloon-Expandable Valve in Low-Risk Patients. N. Engl. J. Med..

[62] Popma J.J., Deeb G.M., Yakubov S.J., Mumtaz M., Gada H., O’Hair D., Bajwa T., Heiser J.C., Merhi W., Kleiman N.S. (2019). Transcatheter Aortic-Valve Replacement with a Self-Expanding Valve in Low-Risk Patients. N. Engl. J. Med..

[63] Synetos A., Ktenopoulos N., Katsaros O., Vlasopoulou K., Drakopoulou M., Koliastasis L., Kachrimanidis I., Apostolos A., Tsalamandris S., Latsios G. (2025). Paravalvular Leak in Transcatheter Aortic Valve Implantation: A Review of Current Challenges and Future Directions. J. Cardiovasc. Dev. Dis..

[64] Yoon S.-H., Bleiziffer S., De Backer O., Delgado V., Arai T., Ziegelmueller J., Barbanti M., Sharma R., Perlman G.Y., Khalique O.K. (2017). Outcomes in Transcatheter Aortic Valve Replacement for Bicuspid Versus Tricuspid Aortic Valve Stenosis. J. Am. Coll. Cardiol..

[65] Patel K.V., Omar W., Gonzalez P.E., Jessen M.E., Huffman L., Kumbhani D.J., Bavry A.A. (2020). Expansion of TAVR into Low-Risk Patients and Who to Consider for SAVR. Cardiol. Ther..

[66] Siontis G.C.M., Jüni P., Pilgrim T., Stortecky S., Büllesfeld L., Meier B., Wenaweser P., Windecker S. (2014). Predictors of permanent pacemaker implantation in patients with severe aortic stenosis undergoing TAVR: A meta-analysis. J. Am. Coll. Cardiol..

[67] Auffret V., Puri R., Urena M., Chamandi C., Rodriguez-Gabella T., Philippon F., Rodés-Cabau J. (2017). Conduction Disturbances After Transcatheter Aortic Valve Replacement. Circulation.

[68] Fadahunsi O.O., Olowoyeye A., Ukaigwe A., Li Z., Vora A.N., Vemulapalli S., Elgin E., Donato A. (2016). Incidence, Predictors, and Outcomes of Permanent Pacemaker Implantation Following Transcatheter Aortic Valve Replacement: Analysis From the U.S. Society of Thoracic Surgeons/American College of Cardiology TVT Registry. JACC Cardiovasc. Interv..

[69] Nuis R.-J., van den Dorpel M., Adrichem R., Daemen J., Van Mieghem N. (2024). Conduction Abnormalities after Transcatheter Aortic Valve Implantation: Incidence, Impact and Management Using CT Data Interpretation. Interv. Cardiol..

[70] Sammour Y., Banerjee K., Kumar A., Lak H., Chawla S., Incognito C., Patel J., Kaur M., Abdelfattah O., Svensson L.G. (2021). Systematic Approach to High Implantation of SAPIEN-3 Valve Achieves a Lower Rate of Conduction Abnormalities Including Pacemaker Implantation. Circ. Cardiovasc. Interv..

[71] Frangieh A.H., Ott I., Michel J., Shivaraju A., Joner M., Mayr N.P., Hengstenberg C., Husser O., Pellegrini C., Schunkert H. (2017). Standardized Minimalistic Transfemoral Transcatheter Aortic Valve Replacement (TAVR) Using the SAPIEN 3 Device: Stepwise Description, Feasibility, and Safety from a Large Consecutive Single-Center Single-Operator Cohort*. Struct. Heart.

[72] Abdelnour M.W., Patel V., Patel P.M., Kasel A.M., Frangieh A.H. (2024). Alternative access in transcatheter aortic valve replacement-an updated focused review. Front. Cardiovasc. Med..

[73] Chandrasekhar J., Hibbert B., Ruel M., Lam B.-K., Labinaz M., Glover C. (2015). Transfemoral vs Non-transfemoral Access for Transcatheter Aortic Valve Implantation: A Systematic Review and Meta-analysis. Can. J. Cardiol..

[74] Elmariah S., Fearon W.F., Inglessis I., Vlahakes G.J., Lindman B.R., Alu M.C., Crowley A., Kodali S., Leon M.B., Svensson L. (2017). Transapical Transcatheter Aortic Valve Replacement Is Associated with Increased Cardiac Mortality in Patients with Left Ventricular Dysfunction: Insights from the PARTNER I Trial. JACC Cardiovasc. Interv..

[75] Di Mario C., Goodwin M., Ristalli F., Ravani M., Meucci F., Stolcova M., Sardella G., Salvi N., Bedogni F., Berti S. (2019). A Prospective Registry of Intravascular Lithotripsy-Enabled Vascular Access for Transfemoral Transcatheter Aortic Valve Replacement. JACC Cardiovasc. Interv..

[76] Généreux P., Piazza N., Alu M.C., Nazif T., Hahn R.T., Pibarot P., Bax J.J., Leipsic J.A., Blanke P., Blackstone E.H. (2021). Valve Academic Research Consortium 3: Updated Endpoint Definitions for Aortic Valve Clinical Research. JACC.

[77] Bapat V.N., Zaid S., Fukuhara S., Saha S., Vitanova K., Squiers J.J., Voisine P., Pirelli L., von Ballmoos M.W., Chu M.W. (2021). Surgical Explantation After TAVR Failure. JACC Cardiovasc. Interv..

[78] Basman C., Pirelli L., Singh V.P., Reimers C.D., Hemli J., Brinster D.R., Patel N.C., Scheinerman S.J., Kliger C.A. (2022). Lifetime management for aortic stenosis: Planning for future therapies. J. Cardiol..

[79] Greenbaum A.B., Kamioka N., Vavalle J.P., Lisko J.C., Gleason P.T., Paone G., Grubb K.J., Bruce C.G., Lederman R.J., Babaliaros V.C. (2021). Balloon-Assisted BASILICA to Facilitate Redo TAVR. JACC Cardiovasc. Interv..

[80] Tang G.H.L., Zaid S., Kleiman N.S., Goel S.S., Fukuhara S., Marin-Cuartas M., Kiefer P., Abdel-Wahab M., De Backer O., Søndergaard L. (2023). Explant vs Redo-TAVR After Transcatheter Valve Failure: Mid-Term Outcomes From the EXPLANTORREDO-TAVR International Registry. JACC Cardiovasc. Interv..

[81] Lee G.S., Tang G., Zaid S., Tam D.Y. (2025). The current state of redo transcatheter aortic valve replacement (TAVR) and limitations: Why TAVR explant is important as the valve reintervention strategy. Ann. Cardiothorac. Surg..

[82] Bowdish M.E., Habib R.H., Kaneko T., Thourani V.H., Badhwar V. (2024). Cardiac Surgery After Transcatheter Aortic Valve Replacement: Trends and Outcomes. Ann. Thorac. Surg..

[83] Fukuhara S., Suzuki T., Deeb G.M., Ailawadi G., Patel H.J., Yang B. (2025). Clinical outcomes of TAVR explant stratified by original risk profile: Insights from 110 TAVR explants. Ann. Cardiothorac. Surg..

[84] SAVR After TAVR Risk Calculator. https://www.sts.org/resources/savr-after-tavr-risk-calculator.

[85] Kaneko T., Vassileva C.M., Englum B., Kim S., Yammine M., Brennan M., Suri R.M., Thourani V.H., Jacobs J.P., Aranki S. (2015). Contemporary Outcomes of Repeat Aortic Valve Replacement: A Benchmark for Transcatheter Valve-in-Valve Procedures. Ann. Thorac. Surg..

[86] de Freitas Campos Guimarães L., Urena M., Wijeysundera H.C., Munoz-Garcia A., Serra V., Benitez L.M., Auffret V., Cheema A.N., Amat-Santos I.J., Fisher Q. (2018). Long-Term Outcomes After Transcatheter Aortic Valve-in-Valve Replacement. Circ. Cardiovasc. Interv..

[87] Pierre D., Arnaud B., Julien H., Thibaud L., Saint E.C., Alizée P., Alexis T., Frederic C., Thierry B., Thomas C. (2020). Transcatheter Valve-in-Valve Aortic Valve Replacement as an Alternative to Surgical Re-Replacement. JACC.

[88] Onorati F., Biancari F., De Feo M., Mariscalco G., Messina A., Santarpino G., Santini F., Beghi C., Nappi G., Troise G. (2015). Mid-term results of aortic valve surgery in redo scenarios in the current practice: Results from the multicentre European RECORD (REdo Cardiac Operation Research Database) initiative†. Eur. J. Cardio-Thorac. Surg. Off. J. Eur. Assoc. Cardio-Thorac. Surg..

[89] Dvir D., Webb J.G., Bleiziffer S., Pasic M., Waksman R., Kodali S., Barbanti M., Latib A., Schaefer U., Rodés-Cabau J. (2014). Transcatheter aortic valve implantation in failed bioprosthetic surgical valves. JAMA.

[90] Allen K.B., Chhatriwalla A.K., Saxon J.T., Huded C.P., Sathananthan J., Nguyen T.C., Whisenant B., Webb J.G. (2021). Bioprosthetic valve fracture: A practical guide. Ann. Cardiothorac. Surg..

[91] Mangieri A., Richter I., Gitto M., Abdelhafez A., Bedogni F., Lanz J., Montorfano M., Unbehaun A., Giannini F., Nerla R. (2024). Chimney Stenting vs BASILICA for Prevention of Acute Coronary Obstruction During Transcatheter Aortic Valve Replacement. JACC Cardiovasc. Interv..

[92] Abdelnour M.W., Zuniga E., Moussa I.D., Patel P.M., Frangieh A.H. (2025). Simulation Training in Cardiovascular Medicine-The Past, Present, and Future: An Updated Comprehensive Overview. Catheter. Cardiovasc. Interv. Off. J. Soc. Card. Angiogr. Interv..

[93] Schultz C.J., Rodriguez-Olivares R., Bosmans J., Lefèvre T., De Santis G., Bruining N., Collas V., Dezutter T., Bosmans B., Rahhab Z. (2016). Patient-specific image-based computer simulation for the prediction of valve morphology and calcium displacement after TAVI with the Medtronic CoreValve and the Edwards SAPIEN valve. EuroIntervention.

[94] Khinsoe G., Ream C., Venkatesh A., Sirset-Becker T., De-Juan-Pardo E.M., Sun Z., Sellers S.L., Leipsic J., Dasi L.P., Ihdayhid A. (2025). CT-derived computational modelling in the lifetime management of aortic stenosis. J. Cardiovasc. Comput. Tomogr..

